# Influence of intergenerational relationships on depressive symptoms in ageing Chinese adults in Hong Kong: Mediating effects of sense of loneliness

**DOI:** 10.1186/s12877-022-03269-z

**Published:** 2022-07-15

**Authors:** Jia-Jia Zhou, Xue Bai

**Affiliations:** 1 Department of Applied Social Sciences, The Hong Kong Polytechnic University, Hong Kong, China; 2 Institute of Active Ageing, The Hong Kong Polytechnic University, Hong Kong, China

**Keywords:** Intergenerational relationships, Sense of loneliness, Depressive symptoms, Affectual closeness, Psychological pathway

## Abstract

**Background:**

Mental health of older adults could be positively predicted by harmonious parent–adult children relationships, although the mechanism has not been sufficiently demonstrated. This study employed sense of loneliness as mediator to examine the influence of multiple domains of intergenerational relationships on depressive symptoms in ageing Chinese adults.

**Methods:**

Data was extracted from a representative survey in Hong Kong among Chinese adults aged over 50 with at least one adult child (*n* = 801). Four key domains (structural–associational, consensual–normative, affectual closeness, and intergenerational conflict) were adopted to measure the intergenerational relationship quality. Depressive symptoms were assessed using the five-item Geriatric Depression Scale. The mediating role of sense of loneliness in the association between intergenerational relationships and depressive symptoms were tested by the PROCESS macro in SPSS.

**Results:**

The influence of overall intergenerational relationship quality and its four subdomains on depressive symptoms were significantly mediated by sense of loneliness. Among the four domains, affectual closeness presented the strongest association with older people’s depressive symptoms. The effect of intergenerational conflict on depressive symptoms was completely mediated by sense of loneliness, and the effects of remaining three domains were partially mediated. The domain of consensual–normative solidarity received the lowest rating by Chinese older adults.

**Conclusion:**

The psychological pathway that loneliness links intergenerational relationships and depressive symptoms was supported in this study. With respect to improving intergenerational relationships, enhancing affectual connection between older parents and adult children was essential to prevent mental problems. This study calls for more attention to the protective role of diverse social relationships in improving mental health through multiple pathways.

## Introduction

Depression is one of the most common mental and psychiatric disorders among older adults [1]. Globally, the prevalence of depression reaches a peak in older adulthood, with a prevalence of 7.5% among women and 5.5% among men [2]. Depressive disorder is a considerable public health concern for older adults in China. It is estimated that the overall prevalence of depressive symptoms among older Chinese people is 20.0% [3]. The burgeoning ageing trend and severe outcomes of late-life depression (e.g., increased mortality rate and suicidal behaviors) [4, 5] have led studies to investigate the multiple risk factors of depression among older adults, including the psychosocial (e.g., social stress), behavioral (e.g., adverse lifestyles), and physiological (e.g., poor physical health) factors [6]. Researchers have discovered that the quality of intergenerational relationships with children plays a critical role in shaping the mental health status of older Chinese adults [7–9].

Derived from Confucianism, the norm of filial piety is culturally expected and practiced cross generations in traditional Chinese society [10]. Under this social norm, adult children have an obligation to provide care for and respect their older parents [11]. Therefore, intergenerational interactions are critical sources of social support for older people, and affectual bonds between older parents and adult children comprise a key kinship network within Chinese families. However, the considerable evolution of Chinese society, such as the narrowing family structure, altered intergenerational living arrangements, and extended educational period of adult children [12], may bring more tensions, disagreements, and pressure to the relations with offspring, threatening older people’s subjective well-being [13]. Therefore, the extent to which and the manner in which intergenerational relationships influence mental health of older adults have novel implications within the context of these transformed social norms.

From the perspective of solidarity [14], conflict [15], and ambivalence [16], the features and structure of intergenerational relationships are characterized by multidimensional properties. Intergenerational relationship quality should not be measured only through binary propositions, but rather multiple aspects, including intergenerational contacts and interactions, similarities, affectual closeness, and intergenerational conflict [17]. However, few studies have employed a multifaceted perspective to evaluate the implications of relationships between older parents and adult children, or employed multiple domains to holistically evaluate intergenerational relationships. Most of the studies have focused only on the influences of intergenerational support and transfers [18–20] and contact frequency [21, 22] on the mental health of older people. To address this gap in the literature, this study will examine various aspects of intergenerational relationships and their associations with depressive symptoms, as well as the linkage pathways.

Notably, existing studies have not sufficiently investigated the pathways through which the multiple domains of intergenerational relations influence the well-being of older parents. Massive evidence has shown that loneliness is a precursor to depressive symptoms among older adults [23–26]. Moreover, in diverse research settings, researchers consistently found that loneliness is a predicted consequence of weak relationships with adult children, such as independent living [27], lack of contact and support from adult children [28], and greater intergenerational ambivalence [29, 30]. Berkman et al. [31] employed a theoretical perspective derived from Durkheim’s work on social integration; they suggested that social ties or social networks may affect mental health through psychological processes. Therefore, as “unfulfilled intimate and social needs”, sense of loneliness may be inferred to be linked to intergenerational relationships and depressive symptoms.

Overall, the associations between the unidimensional aspect of intergenerational relationships and older adults’ mental health have been captured in existing literature. It has been found that intimate emotional bonding between older people and adult children [7–9], receiving financial support [7, 32], close living proximity [7, 33] were related to lower depressive levels and better psychological well-being of older adults. Existing studies have also examined the influence of sense of loneliness in the association between the parent-adult children relationships [27, 27, 29, 30] and mental disorders [23–26]. Yet, the potential mediating role of sense of loneliness in linking the influence of diverse dimensions of intergenerational relationships on older adults’ mental health has not been adequately investigated. To address this limitation, this study primarily aimed to (1) identify the perception of intergenerational relationship quality and its four subdomains (i.e., structural-associational solidarity, consensual-normative solidarity, affectual closeness, intergenerational conflict) among aging Chinese adults; (2) examine the associations between intergenerational relationships (overall and subdomains) and depressive symptoms of older adults respectively; and (3) investigate the psychological pathway that how sense of loneliness mediates the associations between intergenerational relationships (overall and subdomains) and depressive symptoms.

## Conceptual framework and research hypotheses

Building upon the model of intergenerational solidarity [34], intergenerational conflict [15], and intergenerational ambivalence [16], this study highlights the multidimensionality of intergenerational relationships. We used four domains of a well validated scale [17] to comprehensively assess the quality of relationships between Chinese older parents and adult children. The structural-associational domain refers to the residential proximity and frequency of intergenerational interactions; the consensual-normative domain refers to the commonality of the generational opinions or values toward social issues or filial responsibility; affectual closeness refers to the affectional or emotional connection between older parents and adult children; and intergenerational conflict refers to older adults’ tense feelings toward their adult children. These four domains sufficiently presented the profusion and complexity of intergenerational relationships.

**Fig. 1:**
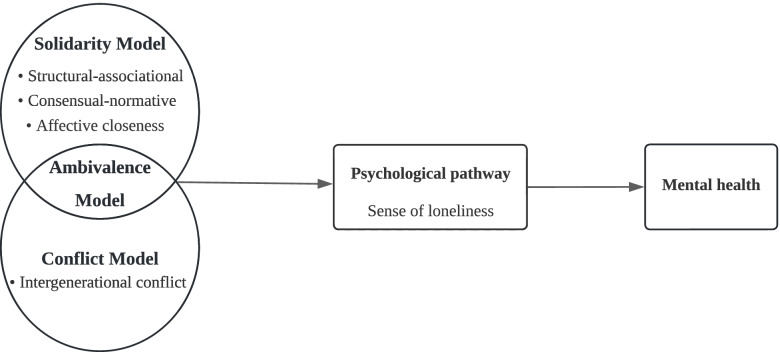
Conceptual framework of psychological pathway linking multiple domains of intergenerational relationships to mental health

The conceptual model of Berkman et al. [31] integrated social networks and health, providing a causal basis for understanding how multiple social relationships influence individual health outcomes. It is argued that social ties shape physical and mental health through psychosocial mechanisms, including social support, social influence, social engagement, and attachment [31]. Within this macro-psychosocial mechanism, loneliness is a proximate mediator of the psychological pathway. In this study, we focus on the influence of relationships between older adults and adult children, which are key kinship ties, on mental health among older people. According to Berkman’s model, the attachment, attitudes, norms, and meaning of social roles resulting from the contact and interaction in intergenerational relationships may reinforce or decrease loneliness in an individual and may, therefore affect older adults’ mental status. Figure 1 presents the overall conceptual framework of the psychological pathway linking the four intergenerational domains to depressive symptoms in older adults.

This study employed sense of loneliness to investigate the psychological pathways linking multiple domains of intergenerational relationships to depressive symptoms. It aimed to investigate the following questions: (1) the extent to which parent–adult children relationships influence depressive symptoms in older people within the context of coexistence of traditional filial piety and transformed social norms, (2) the associations between various domains of intergenerational relationships (i.e., structural-associational solidarity, consensual-normative solidarity, affectual closeness, intergenerational conflict) and depressive symptoms among older adults, and (3) how overall intergenerational relationships and its subdomains influence older adults’ depressive symptoms through sense of loneliness.

Based on previous empirical evidence and conceptual framework, we hypothesised that: (1) poor relationships between older parent and adult children would be positively associated with depressive symptoms in older Chinese; (2) structural-associational solidarity, consensual-normative solidarity, and affectual closeness would be negatively related to mental problems, and intergenerational conflict would be significant predictors to depressive symptoms; and (3) sense of loneliness would mediate the associations between overall and subdomains of intergenerational relationships and depressive symptoms. This study could expand upon and deepen the literature on mental health implications of general intergenerational relationships by concluding specific multidimensional factors. In addition, this study could improve the understanding of psychological pathways linking various intergenerational domains and the mental health of older adults.

## Method

### Participants and sampling

Data from a representative survey, the “Intergenerational Relationship Quality and Care Expectations of Ageing Parents in Hong Kong” were used in this study. Chinese adults aged 50 or older dwelling in Hong Kong and who spoke Cantonese or Mandarin as their primary language were the target population. A two-stage stratified random sampling design was employed. The records were stratified according to geographical district and type of living quarters by using the frame of quarters maintained by the Hong Kong Census and Statistics Department. We adopted a systematic replicated sampling technique with fixed sampling intervals and non-repetitive random numbers to randomly select 5,000 addresses. After excluding vacant, demolished, unidentifiable, and unusable addresses as well as those of individuals ineligible for participation, we obtained a sample size of 1,966. A final valid sample size of 1,001 enabled precise estimates to be determined within the range of ± 3.1 percentage points at the 95% confidence level, indicating that simple random sampling was used.

### Data collection

Trained professional interviewers conducted the face-to-face questionnaire survey between November 2016 and March 2017. Invitation letters stating the research objective were mailed to the selected households. Consent forms were obtained from all participants before the interviews, and the participants were informed of their right to discontinue their participation at any time. Each questionnaire interview lasted approximately 40 min. Computer-assisted personal interviewing and a web support system were used for data collection. Of the 1,966 eligible cases, 1,001 successfully completed the questionnaire, yielding a response rate of 50.9%. For the remaining non-response cases, 76% of cases could not be contacted after more than five attempts of visit in different time periods, and 24% of cases refused to participate in the survey. In accordance with the study purpose, 801 eligible participants with at least one adult child (aged 18 and over) were included for data analysis.

### Measurements

#### Intergenerational relationship quality

Intergenerational relationship quality was measured using the validated Intergenerational Relationship Quality Scale for Aging Chinese Parents (IRQS-AP) [17]. This scale comprises 13 items involving four key relationship domains: structural–associational solidarity, consensual–normative solidarity, affectual closeness, and intergenerational conflict. Measures of structural–associational solidarity included the residential proximity between parents and children (i.e., 1 = living in different cities, 2 = living in the same city but not the same district, 3 = living in the same district but not the same community,4 = living in the same community, and 5 = cohabitation with at least one child); frequency of face-to-face contact and communication by phone, mail, or email, with answers ranging from 1 (very seldom) to 5 (very often); and ageing adults’ assisting their adult children with household chores. Affectual closeness was measured by asking the participants to evaluate their general feelings regarding their closeness with their children, with answers ranging from 1 (not close at all) to 5 (very close); how well they got along with their children; and their frequency of receiving gifts or money from their adult children. Consensual–normative solidarity was assessed according to the participants’ perceived similarity of shared opinions on social and political issues and perceived filial responsibilities regarding care for older relatives. Intergenerational conflict was assessed according to the frequency of tension and strained feelings between ageing parents and their children; the frequency of parents feeling that their children make excessive demands for their help and support or become overly critical of their parents. The Cronbach’s alphas for structural-associational solidarity, affectual closeness, consensual-normative solidarity, and intergenerational conflict subscales were 0.778, 0.758, 0.887, and 0.826 respectively. The total scores of IRQS-AP ranged from 13 to 65, with a higher score indicating higher intergenerational relationship quality. The internal consistency of the scale in our sample, measured using Cronbach’s alpha, was 0.776.

#### Depressive symptoms

Depressive symptoms were assessed using the five-item Geriatric Depression Scale [35]. The five-item short scale of geriatric depression has been proved as a suitable and valid version to measure the precise construction of depression in Chinese context [36, 37]. Participants were asked whether they were satisfied with their lives, felt upset or helpless, would rather stay at home than go out to experience new things, and felt worthless. Total scores ranged from 0 to 5, with a higher score indicating a higher level of depressive symptoms. The scale demonstrated satisfactory internal consistency (Cronbach’s alpha = 0.747) in our sample.

#### Sense of loneliness

The Chinese version of the De Jong Gierveld Six-item Loneliness Scale [38, 39] was used to measure loneliness. The scale consisted of two aspects, namely emotional loneliness and social loneliness. Emotional loneliness was measured by the negative formulated statements: “I experience a general sense of emptiness”; “I miss having people around”; “Often, I feel rejected”. Social loneliness was measured by the positively formulated situations: “There are plenty of people that I can lean on when I have problems”, “There are many people that I can trust completely” and “There are enough people that I feel close to”. Participants responded to the above statements with the following answers: Yes/More or Less/No. Because the answer “more or less” always indicates loneliness [40], for positively formulated items, the neutral and negative answers (“More or less” and “No”) were coded as “1”, and for negatively formulated items, the neutral and positive answers (“More or less” and “Yes”) were coded as “1”. In this way, a higher score indicated a stronger sense of loneliness. By summing up all the dichotomous items, total scores ranged from 0 to 6. The Cronbach’s alpha of sense of loneliness was 0.742.

#### Sociodemographic and health characteristics

Participants were asked to provide information regarding their age, gender (0 = female; 1 = male); employment status (0 = in part-time or full-time employment; 1 = retired or no longer working); education attainment from 1 (illiterate) to 8 (master degree and over); economic status, with answers ranging from 1 (very strained) to 5 (very rich); marital status (1 = married; 0 = separated, widowed or never married); number of children; and functional health status. Respondents’ number of chronic diseases (e.g., heart disease, stroke, hypertension, chronic respiratory disease, asthma, diabetes, etc.) ranged from 0 to 6. Functional health status was assessed by the Lawton Instrumental Activities of Daily Living Scale, which is used to evaluate eight aspects of activities of daily living, including the ability to use the telephone, shop, prepare food, perform housekeeping, do laundry, use transportation, administer medications, and handle finances [41]. IADL scores ranged from 8 to 24, with a higher score indicating poorer functional health status.

### Data analysis

Data analyses were performed using SPSS 26 [42]. Descriptive analyses were conducted first to obtain the mean and standard deviation (SD) for each key study variable, and intercorrelations between study variables were subsequently examined. The mediating role of sense of loneliness in the association between intergenerational relationship quality and each of its four subdomains on depressive symptoms was further tested using the PROCESS macro in SPSS [43]. Age, gender, marital status, employment status, educational attainment, self-perceived economic status, number of children, number of chronic diseases, and IADL scores were included as covariates. The total effect (c), direct effect (c’), and bootstrap-based confidence intervals (CIs) of the indirect effect (ab) were calculated using 5,000 bootstrap samples. Significant mediating effects were indicated by 95% CIs that did not contain zero [44].

## Results

Table 1 lists the socio-demographic characteristics of the participants (*n* = 801). The average age of the participants was 68.58 years, and 43% of the participants were men. Approximately 60% of the participants were married and living with their spouses, whereas others were divorced or separated and widowed or never married. Three-fourths were retired or no longer working, and 25% of them still had part-time or full-time jobs. The average educational attainment (SD) of participants was 2.43 (1.27), which was between primary school and middle school. The mean (SD) of the self-perceived economic status score was 2.95 (0.60). Participants had an average of 2.43 children. The mean (SD) number of chronic diseases was 1.07 (1.12). The mean (SD) score of the 8-item IADL Scale was 8.77 (2.20), indicating that participants had a relatively high ability to live independently.

**Table 1:** Characteristics of participants (*n* = 801)

Demographics or percentage	Mean/Percent	SD
Age	68.58	10.88
Gender (female)	43%	0.50
Marital status (married)	59%	0.49
Employment status (retired)	75%	0.44
Education attainment	2.43	1.27
Self-perceived economic status	2.95	0.60
Number of children	2.43	1.38
Number of chronic diseases	1.07	1.12
Instrumental Activities of Daily Living (IADL) score	8.77	2.20

*Note.* Gender was coded male = 1, female = 0; employment status was coded retired or no longer working = 1, part-time or full-time employment = 0; marital status was coded married = 1, separated, widowed or never married = 0

Table 2 presents the means (SD) and bivariate correlations among the study variables. Compared with other domains, the average score for consensual–normative solidarity (mean = 8.49) was the lowest, indicating that older parents and adult children struggle to achieve similarity in their social values or beliefs in contemporary Hong Kong. This substantial disagreement between generations was also reflected in the perception of intergenerational conflicts (mean = 11.98), with scores indicating that older parents frequently perceive or experience tensions with their adult children. Similarly, scores for affectual closeness were low (mean = 11.01), indicating that parents struggle to get along with and maintain intimate relationships with their adult children. Regarding correlations, the results demonstrated that depressive symptom scores were negatively correlated with overall intergenerational relationship quality (*r* = -0.411) and the four subdomains (consensual-normative solidarity: *r* = -0.226, structural-associational solidarity: *r* = -0.276, affectual closeness: *r* = -0.386, and intergenerational conflict: *r* = -0.164) and were positively associated with sense of loneliness (*r* = 0.462). P-values of all above-mentioned correlations were lower than 0.001.

**Table 2:** Correlations among study variables (*n* = 801)

	Mean (SD)	1	2	3	4	5	6
1. Depressive symptoms	1.04 (1.37)						
2. Intergenerational relationship quality (13–65)	44.63 (6.79)	-.411 ^***^					
3. Consensual-normative solidarity (3–15)	8.49 (2.38)	-.226 ^***^	.658 ^***^				
4. Structural-associational solidarity (4–20)	13.14 (3.61)	-.276 ***	.750 ^***^	.334 ^***^			
5. Affectual closeness (3–15)	11.01 (2.24)	-.386 ^***^	.702 ^***^	.321 ^***^	.376 ^***^		
6. Intergenerational conflict (3–15)	11.98 (2.39)	-.164 ^***^	.397 ^***^	.067	-.065	.169 ^***^	
7. Sense of loneliness	2.70 (1.86)	.481 ^***^	-.462 ^***^	-.213 ^***^	-.235 ^***^	-.436 ^***^	-.336 ^***^

Note. ^***^ *p* < 0.001

**Fig. 2:**
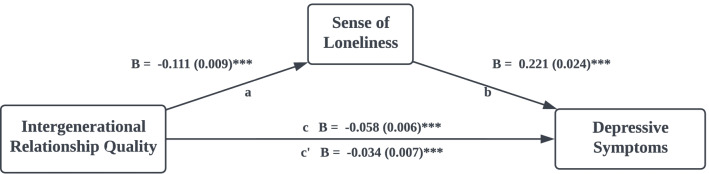
Intergenerational Relationship Quality and Depressive Symptoms Mediated by Sense of Loneliness. *Note.* Age, gender, marital status, employment status, educational attainment, self-perceived economic status, number of children, number of chronic diseases, and IADL score were controlled. Indirect effect (ab): B = -.024, SE = .003, CI 95% = -.031 to -.018. *** *p* < 0.001

A basic mediation model was employed using the PROCESS macro to investigate the role of loneliness in the associations between intergenerational relationships and depressive symptoms. The model (Fig. 2) explained 38.9% (F = 46, *p* < 0.001) of the variance in depressive symptoms after adjustment for age, gender, marital status, employment status, educational attainment, self-perceived economic status, number of children, number of chronic diseases, and IADL scores. Both the total (B = -0.058 (0.006), t = -9.020, CI [-0.071, -0.045]) and direct effects (B = -0.034 (0.007), t = -5.017, CI [-0.047, -0.020]) of overall intergenerational relationship quality on depressive symptoms were significant. Furthermore, sense of loneliness partially mediated this relationship, with the significant indirect effect (B = -0.024 (0.003), CI [-0.031, -0.018]).

**Fig. 3:**
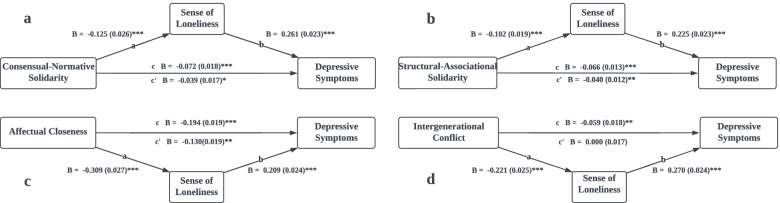
Intergenerational Relationship Sub-Domains and Depressive Symptoms Mediated by Sense of Loneliness. *Note.* Age, gender, marital status, employment status, educational attainment, self-perceived economic status, number of children, number of chronic diseases, and IADL score were controlled. a. Indirect effect (ab): B = -.033, SE = .008, 95% CI = -.048 to -.018. b. Indirect effect (ab): B = -.026, SE = .005, 95% CI = -.037 to -.016. c. Indirect effect (ab): B = -.065, SE = .009, 95% CI = -.084 to -.048. d. Indirect effect (ab): B = -.059, SE = .008, 95% CI = -.077 to -.044. *** *p* < 0.001, ** *p* < 0.01, * *p* < 0.05

Further analyses were performed using the four subdomains of IRQS as independent variables in the mediation model. As presented in Fig. 3, after controlling for aforementioned nine covariates, the total effects of consensual–normative solidarity (B = -0.072 (0.018), t = -3.973, CI [-0.107, -0.036]), structural–associational solidarity (B = -0.066 (0.013), t = -5.132, CI [-0.092, -0.041]), affectual closeness (B = -0.194 (0.019), t = -10.388, CI [-0.231, -0.158]), and intergenerational conflict (B = -0.059 (0.018), t = -3.322, CI [-0.094, -0.024]) on depressive symptoms were all significant. The results also indicated that among the four subdomains of the IRQS, affectual closeness produced a larger influence on mental health among older adults.

The direct effects of consensual–normative solidarity (B = -0.039 (0.017), t = -2.304, CI [-0.072, -0.006]), structural–association solidarity (B = -0.040 (0.012), t = -3.281, CI [-0.064, -0.016]), and affectual closeness (B = -0.130 (0.019), t = -6.709, CI [-0.167, -0.092]) on depressive symptoms were significant, only that of intergenerational conflict (B = 0.000 (0.017), t = -0.013, CI [-0.034, 0.034]) was insignificant and zero. This result suggested that sense of loneliness completely mediated the effect of intergenerational conflict on depressive symptoms (indirect effect: B = -0.059 (0.008), CI [-0.077, -0.044]), indicating that loneliness can fully explain the process through which intergenerational conflict influenced depression [45]. The indirect effects of consensual–normative solidarity (B = -0.033 (0.008), CI [-0.048, -0.018]), structural–associational solidarity (B = -0.026 (0.005), CI [-0.037, -0.016]), and affectual closeness (B = -0.065 (0.009), CI [-0.084, -0.048]) were significant, indicating that loneliness partially mediated the associations between these domains of parents–adult children relationships and depressive symptoms [45].

## Discussion and Implications

This study employed multiple domains of older parents–adult child relationships to systematically investigate the direct and indirect influence of intergenerational relationships on older people’s depressive symptoms through the mediating role of sense of loneliness. All proposed research hypotheses were supported. The associations between the four domains of intergenerational relationships and depressive symptoms were mediated by sense of loneliness. Among the four subdomains, affectual closeness showed a relatively stronger association with depressive symptoms. Regarding the quality of perceived parent–adult child relationships, older adult participants rated consensual–normative solidarity the lowest. The results of this study highlight the importance of harmonious parent–child relationships in preventing mental problems among older adults.

Among the subdomains of intergenerational relationship quality, consensual–normative solidarity received the lowest score. This reflected the divergence of generational attitudes toward social norms and social issues, which was consistent with the findings of a recent survey in Hong Kong where age-related differences in public attitudes towards welfare policy were significant [46]. From the perspective of intergenerational cultural transmission [47], one generation transmits cultural values, beliefs, knowledge, and practices to the next generation. However, the increasing prevalence of individualization, economic independence, and migration [48], have prevented the traditional social norms from being easily transmitted to younger generations. Social values may be altered by generational traits and social experiences that become attached to generationally distinct “collective memories” [49]. Moreover, in a dynamically changing society, such as that of Hong Kong, intergenerational consensus can influence both family harmony and personal well-being, and social stability [50]. Therefore, it is crucial to reinforce mutual understanding between generations at both family and community levels. Service providers could organize intergenerational programs involving both younger and older adults to increase dialogue across generations and improve mutual understanding.

We further found that all four domains of intergenerational relationship quality were significantly associated with depressive symptoms in older adults. This result supplemented the work of Fu and Ji [7] by finding that structural–associational solidarity was also associated with depressive symptoms in Hong Kong older people. Moreover, a perceived intimate affectual connection with adult children demonstrated the strongest association with depressive symptoms, supporting that forming close affectional bonds is a universal human need [51]. Affectual closeness is characterized as a positive emotional sentiment or feeling toward intergenerational solidarity [34], and manifests as intimacy in parent–adult child relationships; getting along well; and providing money or gifts to parents, which are core expectations in traditional filial obligation [10]. In other words, conventional filial expectations remain robust among older Chinese in Hong Kong, and the fulfillment of these core filial norms in the interactions with adult children substantially influences older adults’ loneliness experiences and mental health. As suggested by the study findings, improving the various domains of parent–adult children relationship quality, especially the affectual closeness between generations is pivotal to reducing the risks of suffering depressive symptoms in older people. Policymakers and service providers should promote public education on the strategies of enhancing intergenerational solidarity and provide opportunities for younger generations to more actively engage in intergenerational interactions to strengthen affectual bonding and mutual understanding. Particularly, providing frequent emotional support and financial support to parents are effective ways to improve parents’ mental health, which are highly recommended in the context of declined filial ideology among younger generations [52].

This study also provided evidence pertaining to the mediating effects of loneliness in the association between overall and multiple domains of intergenerational relationships and depressive symptoms among older adults. This result supported the psychological pathway of the conceptual model proposed by Berkman et al. [31], who argued that mental health outcomes could be influenced by social relationships through multiple mechanisms. As a critical constitution of kinship in later life, the quality of intergenerational relationships was remarkably associated with the satisfaction of social and emotional needs among older people, and was further related to their mental health. In addition, this study enriched the understanding of intergenerational relationships by broadly examining the multifaceted components underlying the aforementioned mechanism. It was found that sense of loneliness completely mediated the effect of intergenerational conflict on depressive symptoms and partially mediated the effects of the remaining three dimensions. Undesirable parent–adult child relationships, such as those without children’s support, with reduced interactions, and with intensified conflicts, may initiate and reinforce older adults’ feelings of loneliness, thereby leading to mental problems or affective disorders [53, 54]. Therefore, in addition to improving intergenerational relationships, it would be advisable to encourage older adults, especially those with unsolvable intergenerational conflicts to participate in diverse social activities, such as community-based voluntary activities, physical sessions, and leisure or recreational activities. By doing so, older people can prevent depressive symptoms through broader networks and a reduced sense of loneliness.

### Study limitations

This study has several limitations. Firstly, the characteristics of a cross-sectional study necessitate causal inferences of data analyses to be interpreted with caution; a longitudinal design should be employed to further verify our results. Secondly, the response rate (50.9%) did not reach a high level in this study, which may cause the potential of nonresponse bias [55]. Thirdly, as indicated by the partial mediating effects of loneliness on the associations between the three subdomains of intergenerational relationships (i.e., consensual-normative solidarity, structural-associational solidarity, affectual closeness) and depressive symptoms, alternative mediators may be present in the pathways of the associations identified in this study. In addition to investigating loneliness from a psychological perspective, paths from behavioral and physiological perspectives must be explored in the future studies. Finally, because improving affectual closeness and decreasing loneliness are key to preventing depression in old-age, other sources of intimate relationships, such as romantic partners and close friends, warrant attention in subsequent investigations.

## Data Availability

The datasets used and/or analyzed during the current study available from the corresponding author on reasonable request.
